# Gene expression changes in damaged osteoarthritic cartilage identify a signature of non-chondrogenic and mechanical responses

**DOI:** 10.1016/j.joca.2016.03.007

**Published:** 2016-08

**Authors:** S.L. Dunn, J. Soul, S. Anand, J.-M. Schwartz, R.P. Boot-Handford, T.E. Hardingham

**Affiliations:** † Wellcome Trust Centre for Cell-Matrix Research, University of Manchester, UK; ‡ Stockport NHS Foundation Trust, Manchester, UK

**Keywords:** Cartilage, Osteoarthritis, RNA-seq, Systems biology, PhenomeExpress

## Abstract

**Objectives:**

Joint degeneration in osteoarthritis (OA) is characterised by damage and loss of articular cartilage. The pattern of loss is consistent with damage occurring only where the mechanical loading is high. We have investigated using RNA-sequencing (RNA-seq) and systems analyses the changes that occur in damaged OA cartilage by comparing it with intact cartilage from the same joint.

**Methods:**

Cartilage was obtained from eight OA patients undergoing total knee replacement. RNA was extracted from cartilage on the damaged distal medial condyle (DMC) and the intact posterior lateral condyle (PLC). RNA-seq was performed to identify differentially expressed genes (DEGs) and systems analyses applied to identify dysregulated pathways.

**Results:**

In the damaged OA cartilage, there was decreased expression of chondrogenic genes SOX9, SOX6, COL11A2, COL9A1/2/3, ACAN and HAPLN1; increases in non-chondrogenic genes COL1A1, COMP and FN1; an altered pattern of secreted proteinase expression; but no expression of major inflammatory cytokines. Systems analyses by PhenomeExpress revealed significant sub-networks of DEGs including mitotic cell cycle, Wnt signalling, apoptosis and matrix organisation that were influenced by a core of altered transcription factors (TFs), FOSL1, AHR, E2F1 and FOXM1.

**Conclusions:**

Gene expression changes in damaged cartilage suggested a signature non-chondrogenic response of altered matrix protein and secreted proteinase expression. There was evidence of a damage response in this late OA cartilage, which surprisingly showed features detected experimentally in the early response of cartilage to mechanical overload. PhenomeExpress analysis identified a hub of DEGs linked by a core of four differentially regulated TFs.

## Introduction

Osteoarthritis (OA) is a major cause of pain and disability in older adults, constituting a significant economic burden which is increasing with an ageing population. Current treatments mainly address symptoms and it is important to develop more effective interventions which reduce disease progression^1^. OA is a heterogeneous disease in which there are multiple mechanisms contributing to joint failure, including, misalignment/deformity, muscle weakness, ligament laxity, subchondral bone sclerosis/cysts and osteophyte formation, but one common outcome is cartilage damage and loss^2^.

In OA, the pattern of cartilage damage is typically on the most loaded tibial and femoral surfaces, whilst other less loaded areas remain intact. Previous studies have identified changes in gene expression in OA cartilage and provided evidence that all OA cartilage, including intact cartilage, differs greatly from cartilage on a healthy joint^3^. The changes that accompany OA thus affect all joint cartilage and these changes presumably weaken the tissue such that it becomes damaged and lost at the sites exposed to greatest mechanical load. We therefore set up a study to compare cartilage from intact and damaged sites within the same joint to identify changes in gene expression which may contribute to intact cartilage becoming damaged. This paired analysis of samples from the same joint minimises confounding variables in patient age and genetics and increases the power of the study^4^, ^5^. Identifying genes and regulatory pathways involved as OA cartilage becomes damaged may provide new targets for treatment to delay or reverse the damage.

This study of specific sites of OA knee cartilage was carried out using RNA-sequencing (RNA-seq). Compared to microarray technology this provides a greater dynamic range of analysis with increased sensitivity and specificity to provide enhanced identification of differentially expressed genes (DEGs). We have also applied PhenomeExpress, which incorporates cross-species gene-phenotype associations, to identify dysregulated pathways in damaged OA cartilage.

## Materials and methods

### Study design

Cartilage was obtained under Ethics Committee approval with written informed consent from eight patients with symptomatic OA at total knee replacement (*n* = 8, age range 65–79 years, mean age 70.3). Cartilage from paired osteochondral samples were isolated from the intact posterior lateral condyle (PLC) and the damaged distal medial condyle (DMC) for RNA-seq analysis (Group A). An additional group of paired OA samples were used to validate RNA-seq analysis (*n* = 8, age range 64–76 years, mean age 69.9) (Group B). Cartilage was transferred to RNA later for extraction and RNA-seq and/or reverse transcription quantitative polymerase chain reaction (RT-qPCR) analysis. Full depth osteochondral blocks were taken from adjacent sites, fixed in 10% neutral buffered formalin (Sigma–Aldrich) and decalcified in 20% ethylenediaminetetraacetic acid (EDTA).

### Histology

De-calcified osteochondral samples were dehydrated in graded ethanol (Fisher Scientific) and immersed in xylene (Sigma–Aldrich). Samples embedded in paraffin wax were cut into sections (5 μm thickness) and stained with 0.1% safranin O-fast green for histological grading using a modified Mankin score^6^. Significant differences were determined using one way analysis of variance (ANOVA) non-parametric Friedman test.

### Sulphated glycosaminoglycan assay (sGAG)

The sGAG content of cartilage tissue from the PLC and DMC was determined after overnight digestion in papain at 60°C using the dimethylmethylene blue (DMMB) assay with absorbance read at 570 nm^7^.

### RNA extraction

Total RNA was extracted from 200 to 400 mg of cartilage using TRIzol (LifeTechnologies) reagent and homogenisation (Braun Mikrodismembrator) following freezing in liquid nitrogen. The RNA was purified using RNeasy Qiagen clean-up columns (Qiagen) and for sequencing had a RIN score of >6 (2200 TapeStation, Agilent Technologies).

### RT-qPCR

cDNA was synthesised from 0.5 to 1 μg of total RNA using MLV reverse transcriptase and random hexamers (Life Technologies). For RT-qPCR analysis primer sequences are listed in Supplementary Table 1. Gene expression was normalised to an average of glyceraldehyde 3 phosphate dehydrogenase (GAPDH) and beta actin (ACTB), which were in the bottom 1% of gene variability in the RNA-seq results. Relative gene expression levels were determined using the 2^−ΔΔCt^ analysis method^8^. Differences in expressed genes were identified using the non-parametric Wilcoxon signed-ranked test where *P*-values ≤0.05 were considered significant. Statistical analysis was with GraphPad Prism version 6.04.

### RNA-seq

Strand specific RNA-seq libraries were generated from 0.5 to 1 μg RNA using the TruSeq^®^ Stranded mRNA Sample Preparation Kit (Illumina, Inc.) and 101 bp paired-end reads were generated, yielding at least 39 million reads per sample. The fastq files generated by HiSeq Illumina 2000 platform were analysed with FastQC and scanned against other genomes for possible contamination. Low quality reads, contaminated barcodes and primers were further trimmed with Trimmomatic^9^, ^10^. All libraries were aligned to hg19 assembly of human genome using Tophat-2 with the best score matches reported for each read^11^. The mapped reads were counted by genes with HTSeq against gencode v16 to reflect gene abundance^11^, ^12^. Inter gene expression comparisons were based on calculated fragments per kilobase of transcript per million mapped reads (FPKM). Within the 16 datasets reads from 33,960 (60%) of 56,562 human genes in gencode v16 were detected. Following removal of those with lowest reads, to optimise detection of DEGs, the analysis was on 17,160 genes.

A standard method for estimation of fold change and dispersion for RNA-seq data (DESeq2) was used to initially identify DEGs^13^. The false discovery rate for the analysis (10%) was selected to provide the maximum number of DEG (1575 DEG) with a reasonable level of confidence to best inform the subsequent analysis. For comparison, a lower false discovery rate 5%, gave 1375 DEG (identified in red in Supplementary Table 2, Sheet 2). The 5000 genes with most significant changes by *P*-value were removed and the remaining genes used as *in silico* negative controls for batch effect factor calculation with RUVg^14^. DESeq2 was then used with batch correction to identify DEGs. The resulting *P*-values were adjusted for multiple testing with Benjamini–Hochberg (BH) correction. Data access to R code to reproduce the bioinformatics analysis is at https://github.com/soulj/Dunnetal2015. The RNA-seq data is available from ArrayExpress (E-MTAB-4304).

### Comparison with previous microarray studies

To compare the results with two microarray studies of damaged and intact OA cartilage^4^, ^5^, the dataset GSE57218 was downloaded from Gene Expression Omnibus and Snelling *et al.* provided the raw data from their study^4^, ^5^, ^15^. Both array datasets were analysed as previously reported^4^, ^5^. DEGs in all datasets were defined with 1.5 fold change and an adjusted *P*-value of ≤0.1, which are thresholds used commonly for transcriptomic analysis^16^, ^17^. Hyper-geometric overlap statistics were used to calculate probability of the observed overlap of DEGs.

### PhenomeExpress sub-network identification

PhenomeExpress was used with protein–protein, phenotype–phenotype and protein–phenotype networks created; to identify groups of interacting DEGs related to OA phenotypes^21^. With a maximum initial sub-network size of 7, an empirical *P*-value threshold of 0.05 was used to filter sub-networks by random sampling (10,000 sub-networks) of the filtered PPI network. Phenotypes relevant to OA were chosen on the basis of the web tool Phenomiser and through manual search of the UberPheno ontology (HP:0005086, HP:0001387, MP:0003724 and MP:0003436)^22^.

### Identification of upstream transcription factors (TFs)

The iRegulon framework was used to produce a ranked list of cross-species motif-cluster occurrences in gene promoters as previously described^23^. Briefly, Human promoters (2000 bp upstream, 200 bp downstream of transcriptional start sites) and orthologous regions from Chicken, Chimp, Cow, Dog, Mouse, Rat and Zebrafish were scored using ClusterBuster and then aggregated into a ranked list of target genes for each motif with the RobustRankAggreg Bioconductor package^24^, ^25^. Motifs were then annotated to TFs using a motif-TF network^23^. Chip-seq data from ENCODE and ReMap was used to produce ranked lists of target genes for each TF by the enrichment score^26^, ^27^. Text-mining based TF to gene regulatory interactions were derived from the EVEX database^28^. Coexpression data was taken from CoExpressDB for human experiments across GEO and genes were ranked for each TF by correlation^29^. The differentially expressed upregulated or down-regulated genes were then used to find the enriched upstream TFs using the iRegulon approach of AUC recovery for each motif/TF (z-score ≥3) or using hyper-geometric statistics in the case of the text-mining interactions (BH-adjusted *P*-value ≤0.1). The DEGs predicted to be regulated by these TFs were recovered as previously described^23^.

## Results

### Histology and glycosaminoglycan (GAG) content of the articular cartilage

**Fig. 1:**
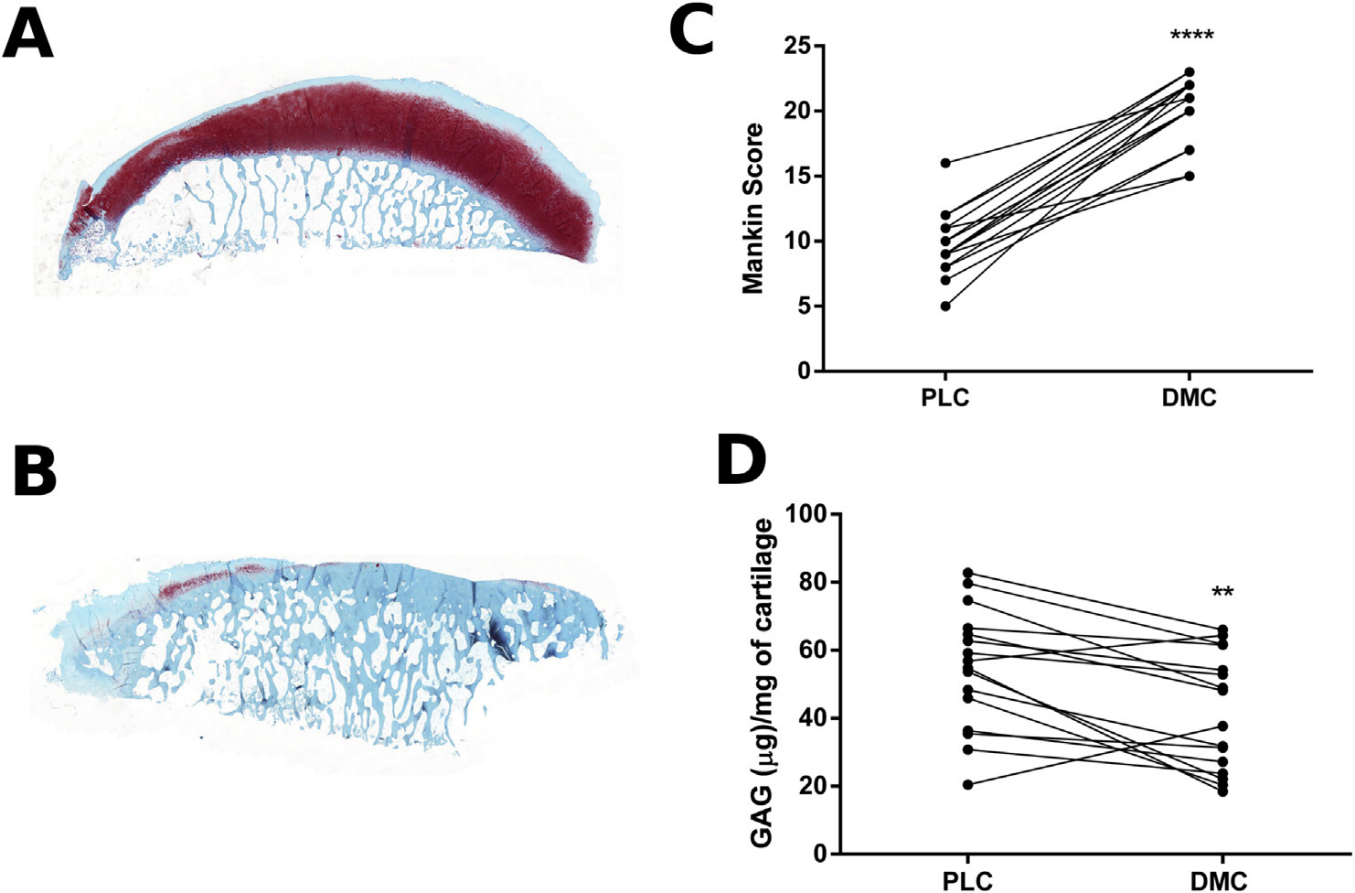
**Histological grading and GAG content of OA articular cartilage**. Safranin O staining of the PLC (A) and DMC (B) of representative OA patient samples. Modified Mankin score of the articular cartilage (C) and GAG content of the cartilage tissue (D) obtained from the PLC and the DMC and used for RNA-seq analysis and an independent patient cohort for validation (*n* = 16). Images acquired using a [*20*×/*0.80 Plan Apo*] objective using the 3D Histech Pannoramic 250 Flash II slide scanner. Magnification× 5. ****P < 0.0001, **P < 0.01.

To characterise the cartilage tissue, the paired OA samples were graded histologically using a modified Mankin score^6^. This confirmed that the cartilage obtained from the DMC (mean ± SD 20.1 ± 0.67) was significantly more damaged than the cartilage obtained from the PLC (9.75 ± 0.62) (*P* < 0.0001) [Fig. 1(A)–(C)]. The GAG content was 19.5% lower in the DMC cartilage (*P* < 0.01) [Fig. 1(D)].

### DEGs

RNA-seq analysis was performed on the eight paired OA DMC and PLC samples and after normalisation and correction for multiple testing we identified 830 genes significantly upregulated and 745 genes significantly down-regulated (4.8 and 4.3% respectively of the total genes analysed) in the damaged cartilage relative to the intact (Supplementary Fig. 1) (full list in Supplementary Table 2). The top upregulated genes with associations to OA or cartilage/chondrocyte biology included; LIF (6.51 fold), TNFAIP6 (TSG-6) (4.67 fold), SERPINE1 (3.94 fold), VCAN (3.46 fold) and WISP1 (3.30 fold) (Supplementary Table 3) and the most down-regulated genes included; TAC1 (−3.35 fold), IGF2 (−2.77 fold) and VIT (−2.43 fold) (Supplementary Table 4).

#### Matrix protein gene expression changes

As the integrity of the matrix is clearly important in OA cartilage, it was noticeable that many extracellular matrix (ECM) genes were amongst the most highly expressed genes in both DMC and PLC samples (22 of the top 50), suggesting both sites have strong matrix production. Comparing the two sites the expression of specific matrix protein genes were increased in the damaged cartilage, but it suggested a change in matrix forming phenotype, as those increased were more associated with non-chondrogenic cells. These included COL1A1 (2.64 fold), FN1 (1.82 fold), COMP (1.48 fold), POSTN (2.99 fold), LAMB3 (3.19 fold), TGFBI (TGFβ induced protein) (2.33 fold) and four highly expressed small leucine rich proteoglycans (SLRPS); LUM (2.21 fold), OGN (2.13 fold), ASPN (2.08 fold) and DCN (1.27 fold), whereas there was decreased expression of chondrocyte associated genes: SOX9 (−1.46 fold), SOX6 (−1.71 fold), ACAN (−1.63 fold), COL9A1 (−2.25 fold), COL9A2 (−1.98 fold) COL9A3 (−1.84 fold), COL11A2 (−2.06 fold) and HAPLN1 (−1.77 fold). The major cartilage collagen COL2A1 was strongly expressed and although there was a small decrease in damaged cartilage, this was not significant. Genes involved in the collagen biosynthesis pathway, LEPREL1 (prolyl 3-hydroxylase2 2.39 fold), P4HA3 (prolyl 4-hydroxylase alpha polypeptide3, 1.92 fold), LOX (1.44 fold lysyl oxidase) and LOXL1 (1.77 fold lysyl oxidase-like 1) were also increased in damaged cartilage (Supplementary Table 2). These ECM protein results suggested a co-ordinated, but not chondrogenic response in the damaged cartilage.

#### Changes in cytokines, proteinases and their inhibitors

The expression of the pro-inflammatory cytokines often associated with OA, including IL-1α/β, IL-6, OSM and TNFα, was barely detected and well below the cut-off used for the analysis (see Methods). There was low but detectable expression of IL-11, which was 2.4 fold increased in damaged and expression of IL-16, but this was unchanged in damaged. Some of the matrix degrading enzymes associated with cartilage degradation in OA such as MMP-1, MMP-13 and ADAMTS-4 were not differentially expressed between damaged and intact sites, but ADAMTS-1 (1.77 fold) −2 (1.47 fold) −5 (2.38 fold), −6 (2.33 fold), −12 (1.81 fold), −14 (3.74 fold) and HTRA1 (2.08 fold) were increased and although TIMP-1 was unchanged, TIMP-2 (1.32 fold), TIMP-3 (1.62 fold) and TIMP-4 (1.41 fold) were all increased. In contrast, MMP-3 (−1.49 fold) gene expression was decreased in the damaged cartilage, as has been previously reported in OA cartilage damage. Results from the RNA-seq data were confirmed by RT-qPCR for selected chondrocyte associated genes, which showed similar results to the RNA-seq in the same eight patients (Group A) and in a separate group of eight additional (Group B) OA patients (Table I).

### Study comparisons

**Fig. 2:**
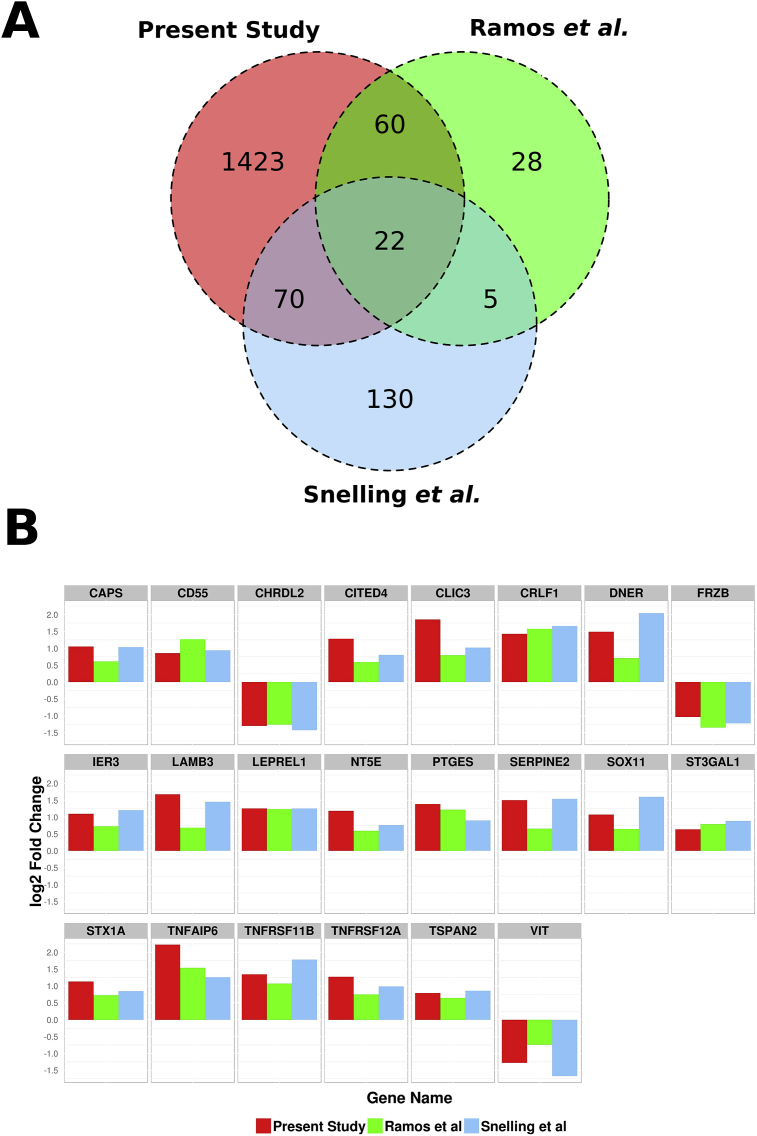
**Comparison of intact vs damaged OA cartilage transcriptome studies**. Overlap of DEGs identified by the RNA-seq data and the two existing microarray datasets Snelling *et al*. and Ramos *et al.* (A). The log2 fold change of the 22 DEGs in the three compared datasets (B).

A comparison of our results with two previous paired damaged and intact OA cartilage studies based on microarrays found statistically significant overlap between the DEGs (3.98 × 10^−37^ and 9.61 × 10^−32^ with the Ramos *et al.* and Snelling *et al.* datasets, respectively). We identified 22 gene changes (fold change ≥1.5, adjusted *P*-value ≤0.1) that were common in all three studies^4^, ^5^ [Fig. 2(A) and (B)]. Genes know to be associated with cartilage biology and OA including CRLF1, PTGES, SERPINE2, TNFAIP6 (TSG-6) and TNFRSF11B (osteoprotegerin) all increased in expression and FRZB and VIT decreased in the expression [Fig. 2(B)]. Individual comparisons between studies are shown in Supplementary Fig. 3.

### Differentially regulated sub-networks

**Fig. 3:**
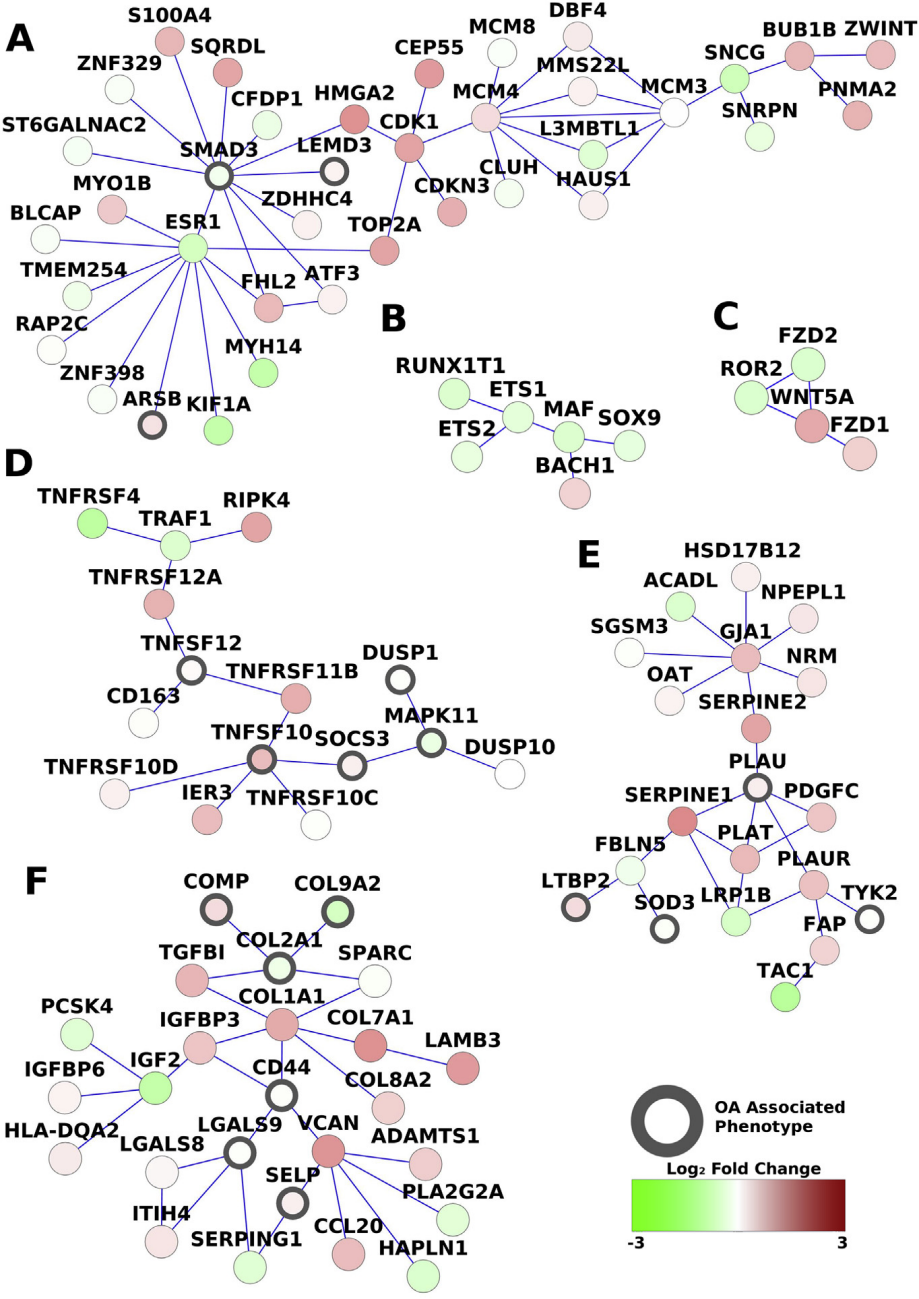
**PhenomeExpress analysis**. Network analysis incorporating cross-species gene-phenotype associations, identified 23 differentially expressed networks based on direct protein–protein interactions in the damaged cartilage. Sub-networks linked to OA included; mitotic cell cycle (*P* = 0.0001) (A), regulation of transcription (*P* = 0.021) (B), Wnt signalling and calcium modulating pathway (*P* = 0.0102) (C), apoptotic processes (*P* = 0.0056) (D), negative regulation of blood coagulation (*P* = 0.0001) (E) and ECM organisation (*P* = 0.0001) (F). The fold change of the proteins is shown by the node colour and OA associated phenotype annotated proteins used to generate the sub-networks are shown with a black border.

We recently described a method, PhenomeExpress, of transcriptomic analysis, in which known gene association with disease is combined with knowledge of protein–protein interaction networks to identify differentially regulated sub-networks enriched in genes associated with the disease phenotype^21^. PhenomeExpress analysis on the paired OA RNA-seq data identified 23 differentially regulated sub-networks enriched in OA phenotype related proteins (Table II). Sub-networks with genes linked to OA included ECM organisation, mitotic cell cycle, regulation of transcription, apoptosis and Wnt signalling [Fig. 3]. The mitotic cell cycle pathway included increases in cell cycle regulatory genes CDK1, CEP55, TOP2A and the alarmin S100A4 [Fig. 3(A)]. The regulation of TF pathway gave further evidence of a decrease in gene expression associated with the chondrocyte phenotype; with less expression of SOX9, ETS1, ETS2 and MAF [Fig. 3(B)]. Altered WNT signalling was identified in the damaged cartilage, with increases in WNT5A and FZD1 and decreases in FZD2 and ROR2 genes [Fig. 3(C)]. Apoptotic processes were found to be dysregulated in the damaged cartilage, with increased expression of multiple members of the tumour necrosis factor receptor superfamily; TNFRSF11B (osteoprotegerin), TNFSF10 (trail or CD253), TNFRSF12A (tweak receptor) and TNFRSF4 (CD134, or OX40) [Fig. 3(D)]. The negative blood coagulation pathway, included the strongly expressed members of the serine proteinase inhibitor superfamily, SERPINE1 (1.98 fold) and SERPINE2 (2.84 fold), but the less expressed proteases, PLAT (tissue plasminogen activator, 2.19 fold) and PLAUR (urokinase, 2.02 fold) were also increased [Fig. 3(E)]. Changes in the ECM organisation pathway were associated with increases and decreases in multiple matrix protein genes as noted above [Fig. 3(F)]. All other PhenomeExpress networks can be found in Supplementary Fig. 2.

### Upstream TFs

**Fig. 4:**
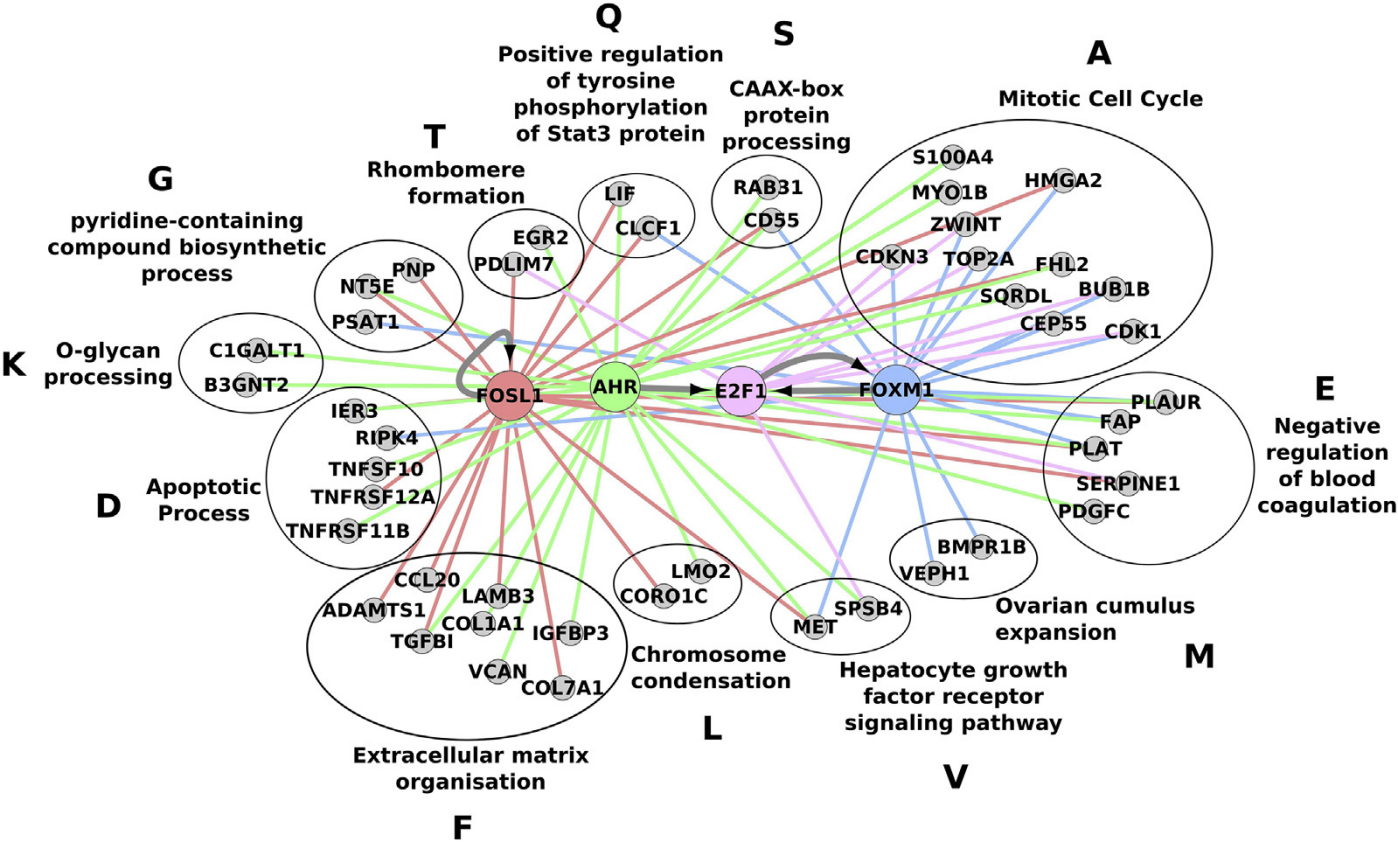
**Multi-layered architecture of damaged OA cartilage signalling**. Network showing regulatory interactions between the differentially expressed, predicted upstream TFs and the PhenomeExpress identified pathways in damaged OA cartilage. Transcriptional links between the four upstream TFs are shown with grey arrows. Regulatory links between the TFs and significantly upregulated target genes present in PhenomeExpress pathways are shown red, green, pink and blue for FOSL1, AHR, E2F1 and FOXM1 respectively. The PhenomeExpress pathways are named by the top enriched GO biological process term. Only PhenomeExpress pathways with at least two target genes present are shown.

We next sought to identify upstream regulators that may explain the observed DEGs and investigate their link to dysregulated biological processes identified with PhenomeExpress. Analysis of the regulatory elements of the DEGs based on (1) TF binding motifs, (2) chip-seq, (3) text-mining and (4) coexpression results identified four TFs (each selected by at least two of the above criteria) controlling their expression (Supplementary Table 5). Further analysis of these four factors (FOXM1, FOSL1, E2F1 and AHR) identified strong regulatory interactions between the PhenomeExpress analysis pathways in damaged OA cartilage (*P* = 6.14 × 10^−25^) [Fig. 4]. Furthermore out of the 830 upregulated genes identified by RNA-seq analysis, 360 were found to be regulated by at least one of the four TFs. Amongst these genes are those with negative associations to chondrocyte biology, including COL1A1, COL7A1, and VCAN, which showed interactions with FOSL1 and AHR; genes in the apoptosis pathway, including tumour necrosis factor receptor superfamily genes; TNFSF10, TNFRSF11B, TNFRSF12A, which showed interactions with AHR and FOXM1 [Fig. 4] and a large proportion of the genes associated with the mitotic cell cycle, which had regulatory links to all four differentially expressed TFs [Fig. 4].

## Discussion

OA is a complex and heterogeneous disease and by investigating the differences between damaged and intact cartilage in a group of OA patients at joint replacement, we hoped to identify active processes that may help explain why damage is progressive and does not lead to successful repair. As the two cartilage sites are in the same joint compartment they are likely to share systemic and locally generated soluble factors, but clearly do not share the same pattern of mechanical load. We therefore propose that the differences between the intact and damaged cartilage reveals how OA cartilage responds to excess load and its consequences as the cartilage is damaged and lost. The changes reported are not therefore interpreted as symptomatic of OA, but they identify the changes in cartilage that underlie its damage.

A major feature of our current RNA-seq analysis is that it helps flesh out the complex pattern of changes in matrix protein gene expression. These changes appear to predict a general decline in differentiated chondrocyte function and the upregulation of matrix genes associated with non-chondrogenic cells. This suggests that the signals governing chondrocyte function in the area of damage are driving responses that are not chondrogenic and do not succeed in stabilising the cartilage matrix to resist further damage. The problem in the damaged cartilage is therefore not a lack of matrix gene expression, but the production of the wrong type of mechanically weak matrix. With the potential to contribute to this response is increased expression of several anabolic growth factors, including high levels of CTGF, which is associated with fibrosis in wound healing and in cartilage matrix remodelling and lesser increases in FGF1, FGF2, TGFB1, TGFB3, BMP2 and BMP6^30^. This growth factor expression might offset the effects of processes such as inflammation from inflammatory cytokines, which have for long been characterised *in vitro* as blocking synthesis and driving degradation of cartilage. However, our analysis in eight OA patients showed that the expression of IL-1α/β, OSM and TNFα was barely detected in either intact or damaged cartilage. This is in general agreement with other studies reporting that these inflammatory cytokines are not expressed in human OA cartilage *in vivo* and from other genome wide analysis, which reports little evidence for a prominent role for them in OA^31^, ^32^, ^33^.

Although these matrix gene expression changes do not appear to be driven by chondrocyte expression of IL-1α/β, OSM or TNFα, there is some evidence of chondrocyte inflammatory response with increased expression of TNFAIP6 (TSG-6), PTGES and iNOS in the damaged cartilage. Whilst the low expression IL-16 and increase in IL-11 may contribute to this, together with the low levels of inflammatory cytokines reported in some OA synovial fluids^34^, ^35^, ^36^, a mechanism that may be more active in the damaged cartilage is the production of various damage-associated molecular pattern (DAMPS) arising from increased proteolytic products of matrix proteins^32^. A leading candidate substrate for DAMPS production is fibronectin, which our results show is very highly expressed and further increased in damaged cartilage and fibronectin fragments are reported to stimulate intracellular S-sulfenylation, which may drive matrix damage pathways^37^. Other possible DAMPS include fragments of fibromodulin, COMP, tenascin C together with aggrecan fragments such as the 32mer, which may all be active in eliciting the increased responses we detect specifically in the cartilage exposed to the greatest load^32^, ^38^.

In assessing which proteinases might be responsible for increased DAMPS production at the DMC site, there was little change in most MMP enzyme expression and furthermore the important MMP inhibitors TIMP2, 3 and 4 were all increased in expression. However, there was some increase in ADAMTS-1 and -5, which both have aggrecanase activity and in ADAMTS-2 and -14, which are procollagen aminopeptidases and in other ADAMTS enzymes with less clear substrates (ADAMTS-6 and -12). There was also an increase in the already highly expressed serine proteinase HTRA1, which is associated with pericellular matrix remodelling in OA animal models^39^. These proteinases may be active in generating DAMPS that drive other downstream changes. This profile of proteinase expression suggests an altered response of the chondrocytes that is quite distinct from that induced by inflammatory cytokines and not historically linked with cartilage degradation. This altered pattern of both matrix gene expression and proteinase expression may be the signature of cartilage damage in the OA joint. It is therefore proposed that a major response of OA cartilage is to remodel matrix abnormally at major loading sites. However, it remains to be determined what causes the changes in gene expression, which are localised to the damaged OA cartilage, to be non-chondrogenic and why they fail to sustain the function of the tissue in the mechanically loaded environment.

The comparison of the results with two other studies on damaged and intact cartilage identified some common changes in a broader human OA context. The study of Snelling *et al.* was on tibial OA cartilage, whereas that of Ramos *et al.* was on hip and knee OA cartilage^4^, ^5^. However, despite the different cartilage sources and analytical methods for the three studies, a core of 22 common gene expression changes were identified, which showed that many changes are common to cartilage at damage sites in different joints and that these studies also detected elements of the OA signature responses, which we now highlight in this study.

PhenomeExpress provides a comprehensive overview of the processes dysregulated in the damaged cartilage of an OA joint using the knowledge gained from natural disease and animal models. Combining this with computational analysis of the promoters of DEGs identified four differentially expressed TFs with links to a large proportion (360 of 830) of DEGs in damaged cartilage. Although they have not previously been studied in OA, E2F1, AHR and the AP1 family member FOSL1 (also known as Fra-1) are known to be involved in chondrocyte differentiation, while FOXM1 is a regulator of the cell cycle^40^, ^41^, ^42^, ^43^. Interestingly, FOSL1 was found to be activated in loaded mandibular cartilage, suggesting its expression may be one of the responses to mechanical load in the DMC^44^. Although, identified as regulating the DEGs independently, these four TFs are also predicted to regulate one another, suggesting a complex regulatory cascade. Furthermore, the target genes of the four TFs had a statistically significant overlap with the genes present in the PhenomeExpress pathways, suggesting these four TFs may play a critical role in regulating the perturbed cellular pathways, such as altered ECM turnover induced in the damaged cartilage.

A limitation of this study is that only eight patients were investigated and therefore it detects only the most common changes. It is also based on transcription and in some cases gene expression may not be tightly linked to protein expression and regulation at the post translational level, such that changes in activity of enzymes such as kinases, or in processes, such as proteinase activation, or proteosomal degradation, may go undetected. It will also not report on factors that may influence gene expression, including microRNAs and mRNA stability^45^. The transcriptomic analysis of chondrocytes thus provides evidence of processes activated and inhibited in damaged cartilage, but it is undoubtedly biased to those with a strong genotypic signature.

Some core elements of the altered pattern of gene expression in damaged OA cartilage are similar to the reported early responses of cartilage to mechanical loading, such as in joint destabilization (DMM) models of OA^46^, ^47^, ^48^. They include early upregulation of Wnt signalling with increased gene expression of several Wnt proteins (5A, 5B, 7B, 9A and 16), increased WISP1 and downstream targets such as osteoprotegerin and down regulation of Wnt antagonists FRZB and DKK1. There was also increases [Fig. 3] associated with cell proliferation, in altered matrix protein expression, particularly in collagen genes and collagen processing enzymes. Chondrocyte response to mechanical loading therefore appears as one of the drivers of responses in damaged OA cartilage. Whether it is the major factor, or just one of many factors responsible for the altered matrix gene and matrix proteinase expression noted above remains to be assessed. What is surprising is that these responses to “loading” of cartilage are still detected at joint replacement and therefore may have persisted in damaged OA cartilage over many years. Much discussion of mechanisms driving cartilage damage in OA has assumed that there are distinct phases that distinguish “early OA” from “late OA”. It is intriguing that some early responses may continue to be active over many years throughout the degenerative process and may therefore provide targets for intervention throughout the clinical progression of OA.

This study identifies from a detailed transcriptional analysis of damaged and intact OA cartilage, evidence for; (1) signature changes in the gene expression of matrix protein and secreted proteinases that may contribute to the progressive damage; (2) changes in late-stage damaged OA cartilage, which are similar to short term responses detected in the DMM mouse model of OA; (3) identification of four TFs in damaged cartilage forming a hub linking multiple regulatory changes identified by PhenomeExpress.

## Authors' contributions

TEH, RBH & JMS conceived the study, secured funding, contributed to its design and co-ordination, participated in interpretation of data and co-wrote the manuscript. SD performed all the laboratory work, participated in interpretation of data and co-wrote the manuscript. JS performed all detailed bioinformatics analyses, participated in interpretation of data and co-wrote the manuscript. SA provided all patient samples, contributed to the study design and co-wrote the manuscript. All authors read and approved the final manuscript.

## Competing interests

None declared.

## Figures and Tables

**Table I:** Real-time PCR validation of RNA-seq analysis. (NS = not significant *P* > 0.05)

Gene name	RNA-seq		Real-time PCR validation of RNA-seq (group A, *n* = 8)		Real-time PCR validation on independent patent cohort (group B, *n* = 8)		Combined real-time PCR validation (group A + B, *n* = 16)	
FC	Adj *P*-val	FC	*P*-val	FC	*P*-val	FC	*P*-val
TNFAIP6	4.67	1.9 × 10^−19^	6.91	7.8 × 10^−03^	2.86	0.02	4.45	<1.0 × 10^−04^
TNFRSF11B	2.54	3.2 × 10^−05^	2.60	0.02	3.13	7.8 × 10^−03^	2.85	<1.0 × 10^−04^
COL1A2	1.52	0.2 (NS)	1.81	7.8 × 10^−03^	1.55	0.3 (NS)	1.67	7.6 × 10^−03^
HAPLN1	−1.77	1.5 × 10^−05^	−2.28	0.02	−2.19	0.02	−2.25	2.0 × 10^−03^
FRZB	−2.05	9.2 × 10^−07^	−2.64	0.02	−3.35	7.8 × 10^−03^	−2.97	2.0 × 10^−03^
COL2A1	−1.29	0.4 (NS)	−1.51	0.06 (NS)	−1.17	0.4 (NS)	−1.33	0.03
ACAN	−1.63	9.3 × 10^−04^	−2.08	7.8 × 10^−03^	−1.47	7.8 × 10^−03^	−1.75	8.0 × 10^−03^
SOX9	−1.46	2.2 × 10^−05^	−1.82	7.8 × 10^−03^	−1.32	0.3 (NS)	−1.55	0.02

**Table II:** **Summary of PhenomeExpress networks**. Differentially regulated sub-networks related to OA phenotypes. The size, empirical *P*-value and the top enriched gene ontology biological process term is indicated for each network

Network number	Network size	Empirical *P*-value	Top GO biological process
A	37	0.0001	Mitotic cell cycle
B	6	0.0214	Positive regulation of transcription from RNA polymerase II promoter
C	4	0.0117	Wnt signalling pathway, calcium modulating pathway
D	15	0.0032	Apoptotic process
E	20	0.0001	Negative regulation of blood coagulation
F	25	0.0001	ECM organization
G	5	0.0228	Pyridine-containing compound biosynthetic process
H	6	0.0014	Anterior/posterior axon guidance
I	7	0.0016	Organ morphogenesis
J	4	0.0087	Regulation of Ras GTPase activity
K	4	0.0143	O-glycan processing
L	8	0.0098	Chromosome condensation
M	4	0.0094	Ovarian cumulus expansion
N	15	0.0002	ER-associated ubiquitin-dependent protein catabolic process
O	6	0.0065	Calcineurin-NFAT signalling cascade
P	7	0.0134	Acetylcholine secretion, neurotransmission
Q	8	0.0006	Positive regulation of tyrosine phosphorylation of Stat3 protein
R	8	0.0129	Regionalization
S	6	0.049	CAAX-box protein processing
T	7	0.008	Rhombomere formation
U	5	0.0196	Positive regulation of receptor recycling
V	5	0.0114	Hepatocyte growth factor receptor signalling pathway
W	5	0.0089	Response to lipopolysaccharide
