# Supplementary material for: Gene expression changes in damaged osteoarthritic cartilage identify a signature of non-chondrogenic and mechanical responses
Source: Osteoarthritis Cartilage. 2016 Aug;24(8):1431–40. doi: 10.1016/j.joca.2016.03.007 (PMC4989048; doi:10.1016/j.joca.2016.03.007)
Supplement: Supplementary file 1 [file mmc1.pdf]

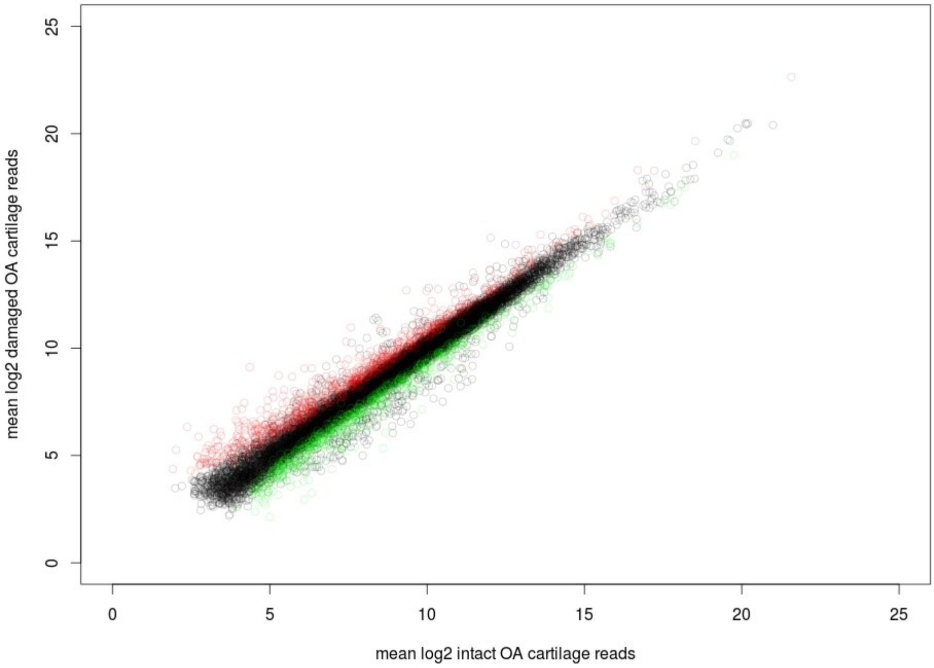

Supplementary Figure 1. Log2 RNA-seq reads of intact vs damaged cartilage. Of the 17160 genes included in the analysis, 4.8% were significantly up-regulated (red) while 4.3% were significantly down-regulated (green). (fold change ≥1.5, adjusted p-value ≤0.1).

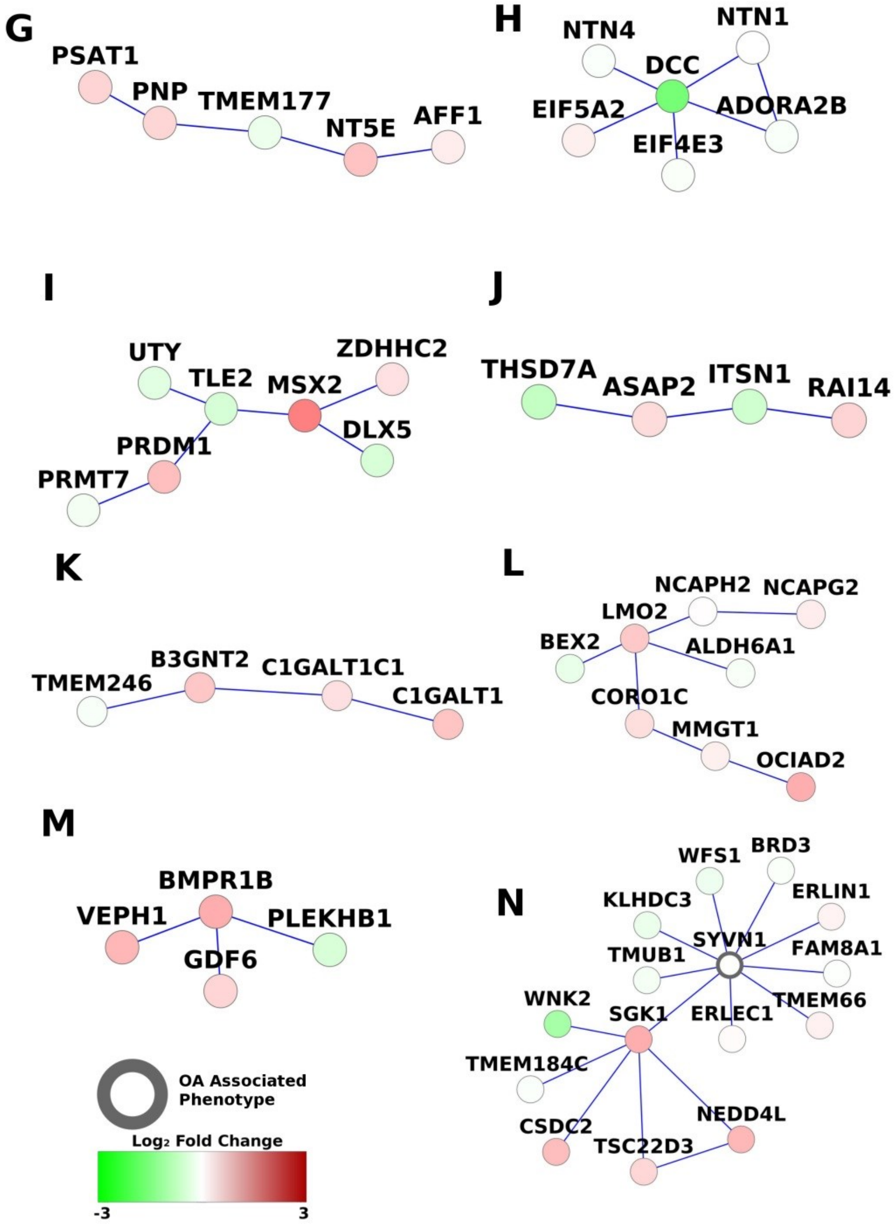

Supplementary Figure 2. PhenomeExpress Analysis. Network analysis incorporating cross-species gene-phenotype associations, identified 23 differentially expressed networks based on direct protein-protein interactions in the damaged cartilage. Sub-networks included; pyridine-containing compound biosynthetic **(G)**, process anterior/posterior axon guidance **(H)**, organ morphogenesis **(I)**, regulation of Ras GTPase activity **(J)**, O-glycan processing **(K)**, chromosome condensation **(L)**, ovarian cumulus expansion **(M)**, ER-associated ubiquitin-dependent protein catabolic process **(N).** The fold change of the proteins is shown by the node colour and OA associated phenotype annotated proteins used to generate the sub-networks are shown with a black border.

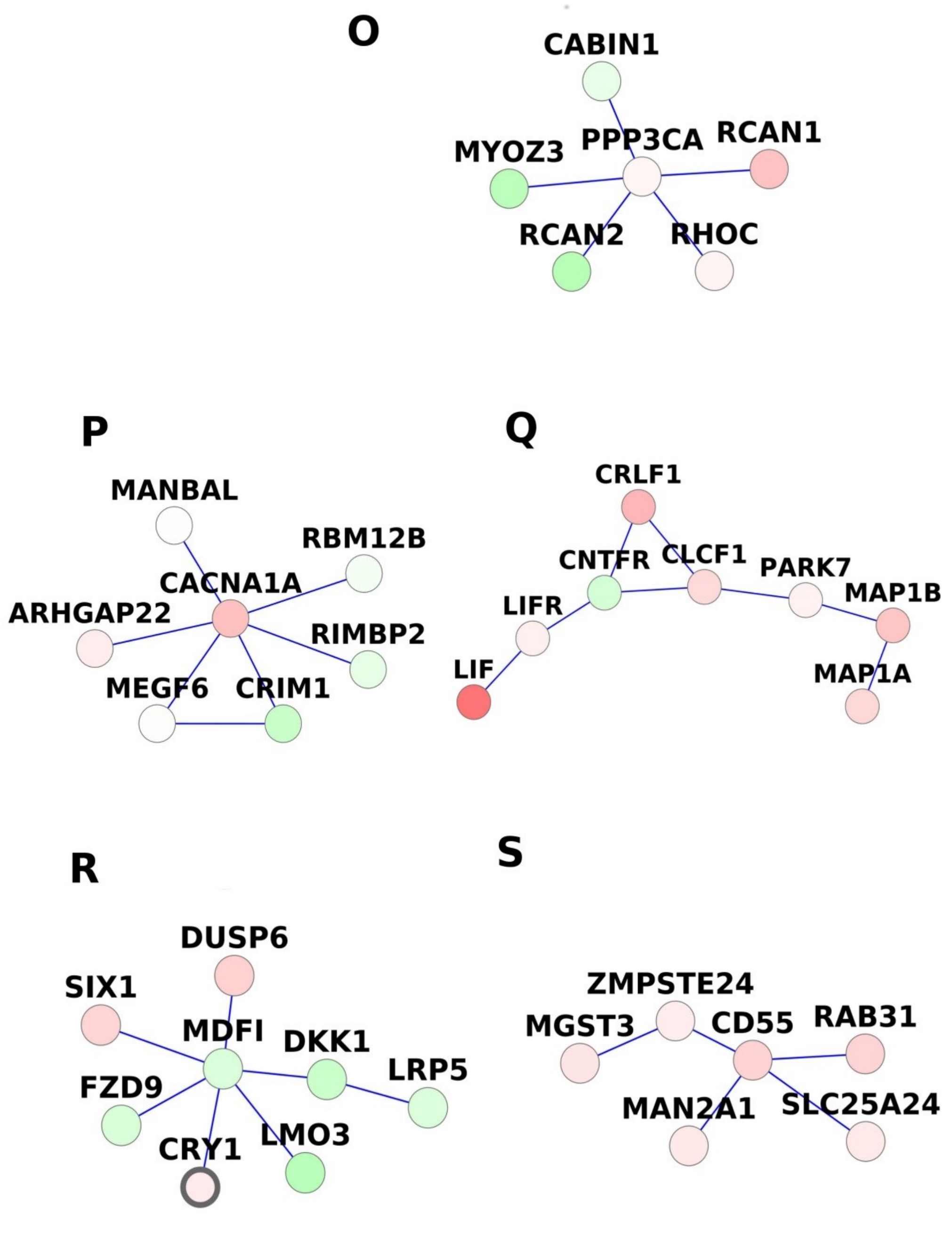

Supplementary Figure 2 Cont. PhenomeExpress Analysis. calcineurin-NFAT signaling cascade **(O)**, acetylcholine secretion **(P)**, neurotransmission **(Q)**, positive regulation of tyrosine phosphorylation of Stat3 protein **(R)**, regionalization, CAAX-box protein processing **(S)**.

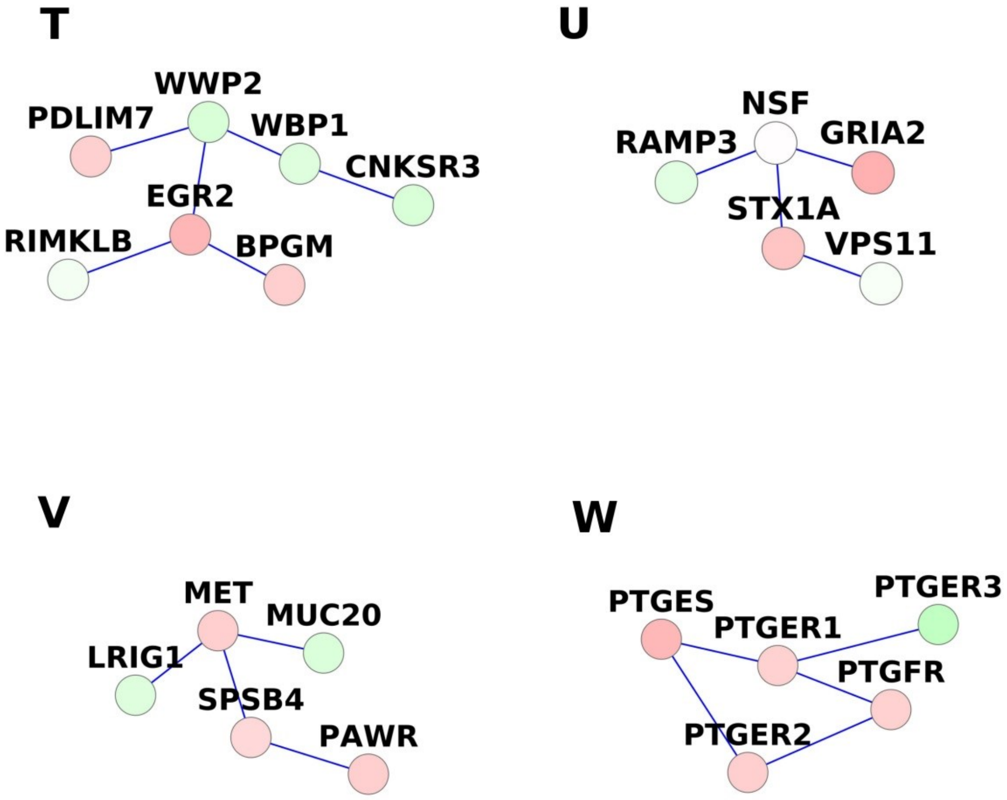

Supplementary Figure 2 Cont. PhenomeExpress Analysis. rhombomere formation **(T)**, positive regulation of receptor recycling **(U)**, hepatocyte growth factor receptor signaling pathway **(V)**, response to lipopolysaccharide **(W)**.

A

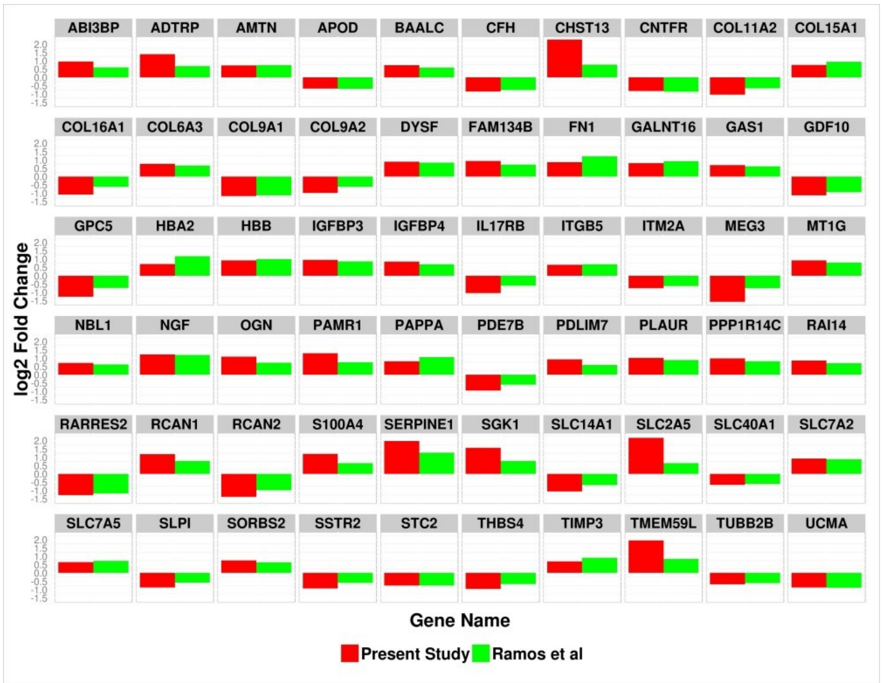

B

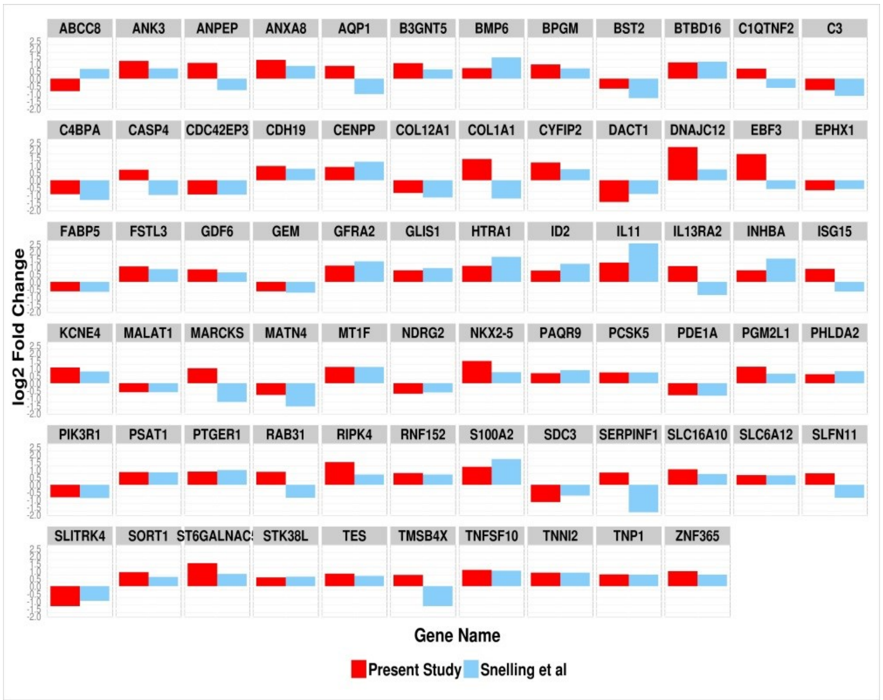

Supplementary Figure 3. Comparison of intact vs damaged OA cartilage transcriptome studies. The log2 fold change of the differentially expressed genes in the three comparable datasets, the present study compared to Ramos *et al* **(A)** and Snelling *et al* **(B)**.

Supplementary Table 1. Primer sequences used in real-time PCR for RNA-seq validation.

Gene	Forward Primer	Reverse Primer
GAPDH	5’-AGGGCTGCTTTTAACTCTGGT-3’	5’-CCCCACTTGATTTTGGAGGGA-3’
ACTB	5’-CCACCATGTACCCAGGCATT-3’	5’-CACATCTGCTGGAAGGTGGA-3’
COL1A2	5’-GGCTACCCAACTTGCCTTCA-3’	5’GCAGTGGTAGGTGATGTTCTGAGA3’
COL2A1	5’GGCAATAGCAGGTTCACGTACA-3’	5’-CGATAACAGTCTTGCCCCACTT-3’
ACAN	5’-TCGAGGACAGCGAGGCC-3’	5’-TCGAGGGTGTAGCGTGTAGAGA-3’
SOX9	5’-GACTTCCGCGACGTGGAC-3’	5’-GTTGGGCGGCAGGTACTG-3’
TNFAIP6	5’-CTCCATATGGCTTGAACGAGCAGC-3’	5’-GCCCTTAGCCATCCATCCAGCA-3’
TNFRSF11B	5’-TCCTGGATTTGGAGTGGTGC-3’	5’-CTCCTCACACAGGGTAACATCTAT-3’
HAPLN1	5’-ACTTGAGAGCATCCGAACTCC-3’	5’-GGGGGCCATTTTCTGCTTG-3’

Supplementary Table 3. The top 50 up-regulated genes in damaged cartilage relative to intact cartilage

Gene Name	Gene Symbol	FC	Adjusted p-value
Family with sequence similarity 167, member A	FAM167A	7.49	$1.29x10^{-34}$
Leukemia inhibitory factor	LIF	6.51	$1.24x10^{-21}$
Msh homeobox 2	MSX2	5.62	$2.11x10^{-19}$
Carbohydrate (chondroitin 4) sulfotransferase 13	CHST13	4.83	$1.67x10^{-12}$
Tumor necrosis factor, alpha-induced protein 6	TNFAIP6	4.67	$1.85x10^{-19}$
DnaJ (Hsp40) homolog, subfamily C, member 12	DNAJC12	4.63	$2.95x10^{-13}$
RP11-267A15.1	RP11-267A15.1	4.50	$5.17x10^{-12}$
Solute carrier family 2 (facilitated glucose/fructose transporter), member 5	SLC2A5	4.49	$1.27x10^{-35}$
RP11-536I6.2	RP11-536I6.2	4.16	$3.81x10^{-10}$
Forkhead box F1	FOXF1	3.98	$2.41x10^{-11}$
Serpin peptidase inhibitor, clade E (nexin, plasminogen activator inhibitor type 1), member 1	SERPINE1	3.94	$1.81x10^{-24}$
Transmembrane protein 59-like	TMEM59L	3.89	$1.41x10^{-11}$
Phosphatidylinositol-3,4,5-trisphosphate-dependent Rac exchange factor 2	PREX2	3.87	$1.85x10^{-19}$
Teneurin transmembrane protein 3	TENM3	3.78	$6.89x10^{-16}$
Neurotrophin 3	NTF3	3.77	$3.54x10^{-12}$
ADAM metallopeptidase with thrombospondin type 1 motif, 14	ADAMTS14	3.74	$1.56x10^{-14}$
Anillin, actin binding protein	ANLN	3.72	$3.32x10^{-17}$
Urocanate hydratase 1	UROC1	3.67	$6.29x10^{-14}$
Protocadherin 10	PCDH10	3.66	$9.41x10^{-12}$
Chloride intracellular channel 3	CLIC3	3.62	$5.27x10^{-13}$
Collagen, type VII, alpha 1	COL7A1	3.59	$1.51x10^{-14}$
High mobility group AT-hook 2	HMGA2	3.58	$6.00x10^{-09}$
Creatine kinase, brain	CKB	3.57	$5.55x10^{-19}$
AC093850.2	AC093850.2	3.52	$2.34x10^{-09}$
Target of myb1 (chicken)-like 1	TOM1L1	3.50	$1.93x10^{-11}$
Hes-related family bHLH transcription factor with YRPW motif 2	HEY2	3.49	$3.86x10^{-13}$
Dual specificity phosphatase 4	DUSP4	3.48	$1.38x10^{-13}$
Versican	VCAN	3.46	$6.97x10^{-18}$
Eva-1 homolog A (C. elegans)	EVA1A	3.38	$7.24x10^{-08}$
Early B-cell factor 3	EBF3	3.33	$1.58x10^{-07}$
Leucine rich repeat containing 8 family, member E	LRRC8E	3.32	$8.84x10^{-11}$
WNT1 inducible signaling pathway protein 1	WISP1	3.30	$1.85x10^{-19}$
FAT atypical cadherin 3	FAT3	3.28	$4.32x10^{-10}$
Laminin, beta 3	LAMB3	3.19	$4.93x10^{-13}$
Centrosomal protein 55kDa	CEP55	3.18	$2.20x10^{-07}$
Solute carrier family 38, member 5	SLC38A5	3.11	$1.16x10^{-22}$
Hormonally up-regulated Neu-associated kinase	HUNK	3.11	$4.78x10^{-11}$
RP11-160A10.2	RP11-160A10.2	3.11	$8.74x10^{-08}$
Family with sequence similarity 132, member B	FAM132B	3.05	$3.04x10^{-06}$
Carbonic anhydrase XII	CA12	3.03	$6.11x10^{-08}$
SLIT and NTRK-like family, member 6	SLITRK6	3.03	$3.96x10^{-06}$
T-box 3	TBX3	3.03	$7.84x10^{-07}$
Bone morphogenetic protein receptor, type IB	BMPR1B	3.01	$2.16x10^{-06}$
Potassium voltage-gated channel, delayed-rectifier, subfamily S, member 3	KCNS3	2.99	$4.56x10^{-09}$
Periostin, osteoblast specific factor	POSTN	2.99	$2.38x10^{-06}$
Serum/glucocorticoid regulated kinase 1	SGK1	2.97	$6.71x10^{-25}$
OCIA domain containing 2	OCIAD2	2.97	$3.16x10^{-15}$
Fibroblast growth factor 9	FGF9	2.94	$1.62x10^{-06}$
Non-SMC condensin I complex, subunit G	NCAPG	2.94	$3.36x10^{-06}$
Cyclin-dependent kinase 17	CDK1	2.93	$5.66x10^{-07}$

Supplementary Table 4. The top 50 down-regulated genes in damaged cartilage relative to intact cartilage

Gene Name	Gene Symbol	FC	Adjusted p-value
Deleted in colorectal carcinoma	DCC	6.37	1.60×10^{-25}
Transmembrane protein 176A	TMEM176A	3.72	1.96×10^{-12}
Tachykinin, precursor 1	TAC1	3.35	4.26×10^{-12}
CD52 molecule	CD52	3.33	1.25×10^{-08}
WNK lysine deficient protein kinase 2	WNK2	3.30	5.55×10^{-19}
Angiotensin II receptor, type 2	AGTR2	3.22	8.01×10^{-07}
Leucine rich repeat transmembrane neuronal 2	LRRTM2	3.09	1.40×10^{-11}
Tumor necrosis factor receptor superfamily, member 4	TNFRSF4	3.07	1.31×10^{-08}
Even-skipped homeobox 1	EVX1	3.06	3.24×10^{-06}
Chromosome 1 open reading frame 173	C1orf173	3.04	4.90×10^{-07}
Solute carrier family 22	SLC22A11	3.01	6.91×10^{-08}
Maternally expressed 3	MEG3	2.99	8.80×10^{-14}
Calcium channel, voltage-dependent, alpha 2/delta subunit 2	CACNA2D2	2.98	3.58×10^{-17}
Neurexin 2	NRXN2	2.94	7.82×10^{-10}
Calpain 6	CAPN6	2.90	4.15×10^{-06}
Seizure related 6 homolog (mouse)-like 2	SEZ6L	2.90	4.15×10^{-08}
Neurotrophic tyrosine kinase, receptor, type 3	NTRK3	2.89	1.21×10^{-05}
3-hydroxymethyl-3-methylglutaryl-CoA lyase-like 1	HMGCLL1	2.83	1.84×10^{-07}
Insulin-like growth factor 2 mRNA binding protein 2	IGF2	2.77	4.17×10^{-18}
NFAT activating protein with ITAM motif 1	NFAM1	2.74	4.49×10^{-10}
Transmembrane protein 176B	TMEM176B	2.73	1.89×10^{-06}
Family with sequence similarity 101, member A	FAM101A	2.73	2.37×10^{-09}
Mesothelin	MSLN	2.73	1.24×10^{-05}
Family with sequence similarity 198, member A	FAM198A	2.72	1.78×10^{-11}
Dishevelled-binding antagonist of beta-catenin 1	DACT1	2.72	2.83×10^{-18}
Sarcalumenin	SRL	2.70	8.36×10^{-06}
Zinc finger protein 385C	ZNF385C	2.68	3.58×10^{-13}
Myosin, heavy chain 14, non-muscle	MYH14	2.68	1.04×10^{-09}
Kinesin family member 1A	KIF1A	2.66	2.09×10^{-07}
Cadherin-related family member 1	CDHR1	2.65	6.98×10^{-08}
R-spondin 3	RSPO3	2.65	1.73×10^{-06}
Regulator of calcineurin 2	RCAN2	2.61	1.23×10^{-08}
RP11-588K22.2	RP11-588K22.2	2.59	4.78×10^{-10}
ALX homeobox 4	ALX4	2.58	7.28×10^{-07}
Small G protein signaling modulator 1	SGSM1	2.58	3.05×10^{-08}
LIM domain only 3 (rhombotin-like 2)	LMO3	2.57	1.61×10^{-05}
Guanylate cyclase 1, soluble, alpha 3	GUCY1A3	2.56	1.73×10^{-14}
PR domain containing 16	PRDM16	2.55	3.18×10^{-05}
Myozenin 3	MYOZ3	2.53	5.39×10^{-13}
Long intergenic non-protein coding RNA 284	LINC00284	2.53	4.26×10^{-07}
Glutamate receptor, ionotropic, N-methyl D-aspartate 2C	GRIN2C	2.52	2.22×10^{-07}
Serine/threonine kinase 32B	STK32B	2.51	6.71×10^{-25}
SLIT and NTRK-like family, member 4	SLITRK4	2.50	6.55×10^{-08}
Cardiomyopathy associated 5	CMYA5	2.48	3.94×10^{-10}
Chordin-like 2	CHRDL2	2.47	7.69×10^{-10}
Piezo-type mechanosensitive ion channel component 2	PIEZO2	2.45	5.70×10^{-21}
RP11-561C5.3	RP11-561C5.3	2.43	4.01×10^{-05}
Kyphoscoliosis peptidase	KY	2.43	1.04×10^{-04}
Vitrin	VIT	2.43	3.19×10^{-04}
Retinoic acid receptor responder	RARRES2	2.42	1.52×10^{-06}

Supplementary Table 5. Upstream Transcription factor analysis. Motif, Chip-Seq, text-mining and coexpression based methods were used to identify possible upstream transcription factors (TFs) controlling the expression of the up-regulated differentially expressed genes in damaged OA cartilage. For each TF detected by at least 2 methods the z-score or BH-adjusted p-value is shown for each tool as is the fold-change and p-value of the TF. TFs not included in a method are shown by NA while non-significant z-scores and p-values are shown by NS. Significantly differentially expressed TFs are highlighted in bold.

Gene Symbol	FC	padj	Motif z-Score	Chip-Seq z-Score	Textmining padj	Coexpression z-Score
**AHR**	1.75	0.01	NS	NS	0.02	3.71
BMP2	1.18	0.28	NA	NA	9.48×10^{-04}	4.83
BRCA1	1.32	0.16	NS	NS	4.76×10^{-04}	3.10
CTCF	-1.05	0.71	NS	4.29	8.43×10^{-03}	NS
DNMT3B	1.28	0.45	NA	NA	0.03	3.34
**E2F1**	1.78	0.01	NS	4.01	3.39×10^{-04}	3.11
E2F4	-1.06	0.67	NS	3.95	3.39×10^{-04}	NS
EZH2	1.50	0.11	NA	3.12	0.02	3.31
FOS	-1.02	0.96	4.94	NS	0.03	3.74
FOSB	-1.07	0.87	4.94	NA	NS	3.55
**FOSL1**	1.62	0.04	4.94	NS	NS	3.46
**FOXM1**	2.35	1.38×10^{-04}	3.14	3.47	1.24×10^{-03}	3.01
HES1	-1.20	0.24	4.21	NA	1.58×10^{-03}	NS
HOXA10	-1.26	0.04	3.39	NA	4.76×10^{-04}	NS
ID3	-1.09	0.70	NA	NA	7.27×10^{-03}	3.40
JUN	1.11	0.80	4.05	NS	2.50×10^{-03}	NS
JUNB	1.03	0.92	4.94	NS	0.04	3.19
JUND	1.10	0.73	4.94	NS	0.03	NS
SNAI2	-1.36	0.03	NS	NA	0.06	4.82
ZBTB16	-1.09	0.78	NS	NA	2.97×10^{-03}	3.68
